# Supplementary figures and images for: Selection of stimulus parameters for enhancing slow wave sleep events with a neural-field theory thalamocortical model
Source: PLoS Comput Biol. 2021 Jul 30;17(7):e1008758. doi: 10.1371/journal.pcbi.1008758 (PMC8357165; doi:10.1371/journal.pcbi.1008758)

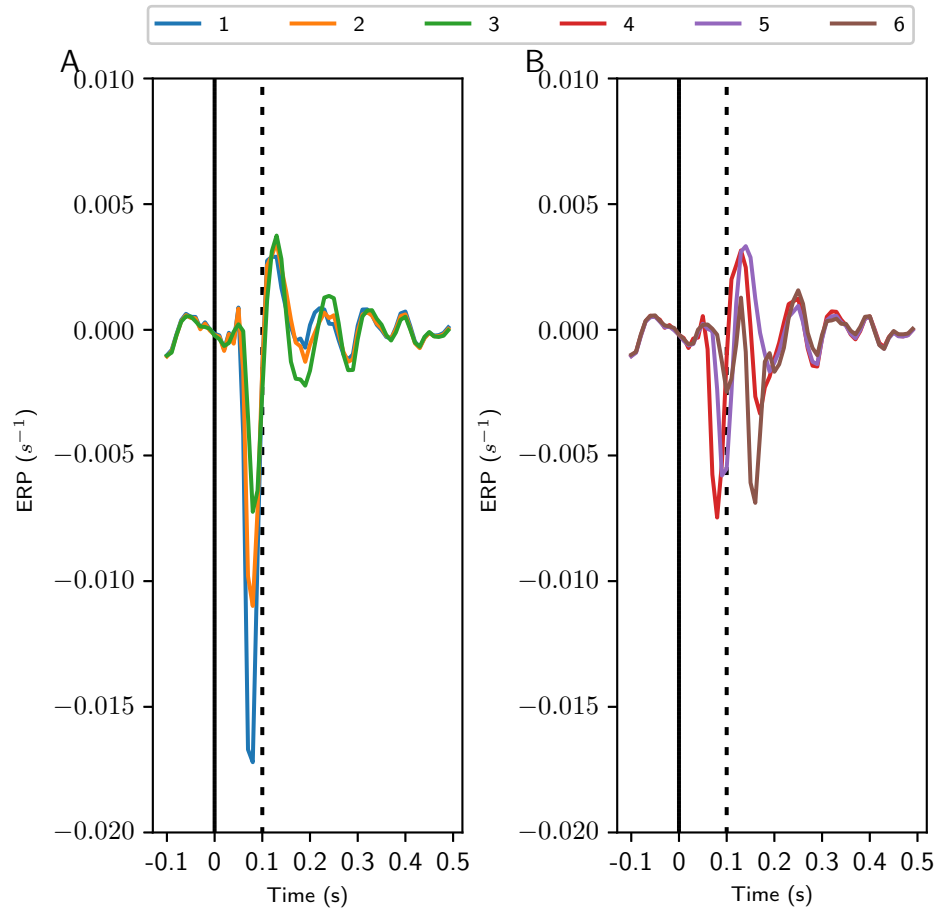

Supplement: S1 Fig — Average activity of the cortical activity from simulations with different stimulus shapes. The subtracted baseline is selected from -450 ms to the stimuli onset. The stimulus onset is marked with the bold vertical line and the stimulus turnoff is marked with the dashed line. Shapes: (A) decreasing ramp (1), rectangular trapezoid (2), Gaussian (3); (B) rectangular (4), triangular (5), and rising ramp (6). (PDF) [file pcbi.1008758.s001.pdf]

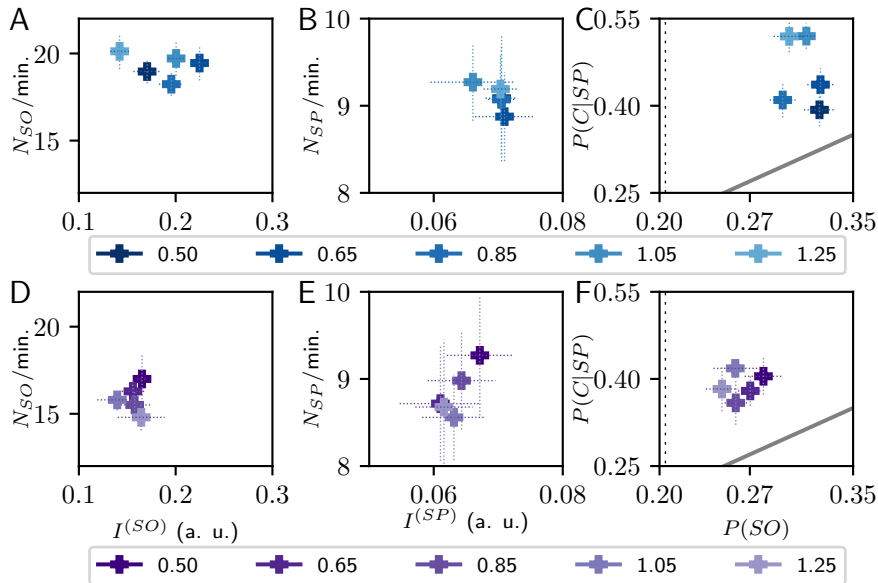

Supplement: S2 Fig — (A) Changes by stimulation frequency on SO measurements. (B) Changes by stimulation frequency on spindles measurements. (C) Changes by stimulation frequency on probabilities of coincident events. (D) Changes by different mean λ of random stimulation on SO measurements. (E) Changes by different mean λ of random stimulation on spindles measurements. (F) Changes by different mean λ of random stimulation on probabilities of coincident events. (PDF) [file pcbi.1008758.s002.pdf]

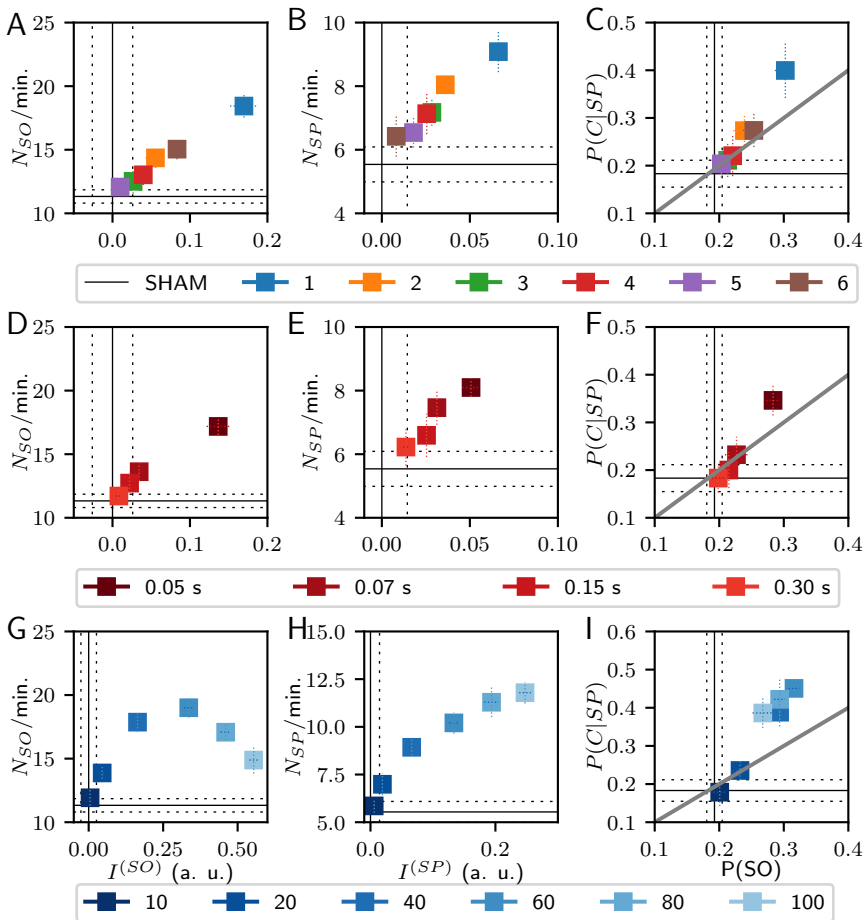

Supplement: S3 Fig — Top:(A) Changes by shape in SO. (B) Changes by shape in spindles. (C) Changes in the probability of co-occurrence by shape. (D) Changes by pulse duration in SO. (E) Changes by pulse duration in spindles. (F) Changes by pulse duration in the probability of co-occurrence. (G) Changes by pulse energy in SO. (H) Changes by pulse energy in spindles. (I) Changes by pulse energy in the probability of co-occurrence. (PDF) [file pcbi.1008758.s003.pdf]

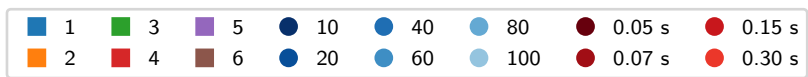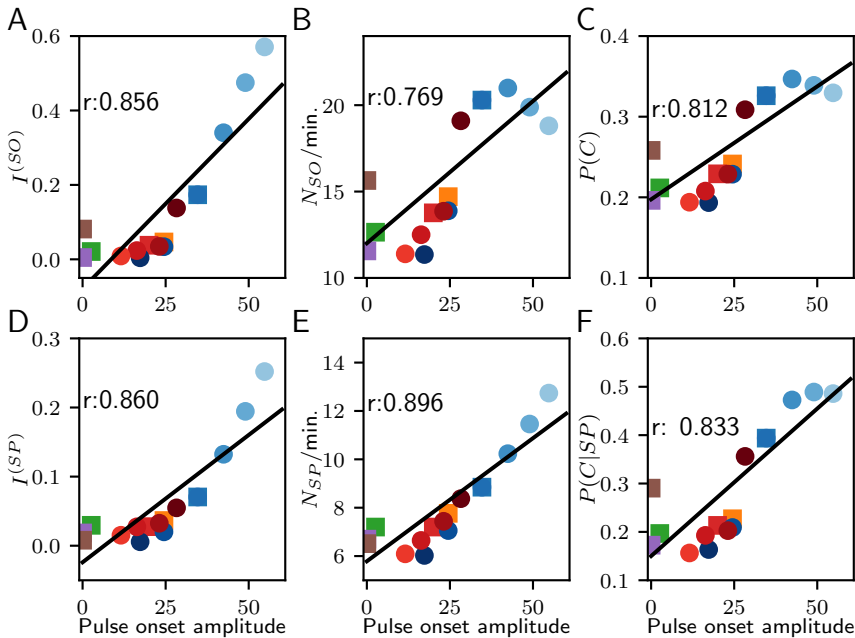

Supplement: S4 Fig — (A) Onset amplitude of stimulus vs I(SO). (B) Onset amplitude of stimulus vs slow oscillations. (C) Onset amplitude of stimulus vs probability of co-occurrence of events. (D) Onset amplitude of stimulus vs I(SP). (E) Onset amplitude of stimulus vs spindles. (F) Onset amplitude of stimulus vs conditional probability of co-occurrence respect to the occurrence of spindles. (PDF) [file pcbi.1008758.s004.pdf]

**A**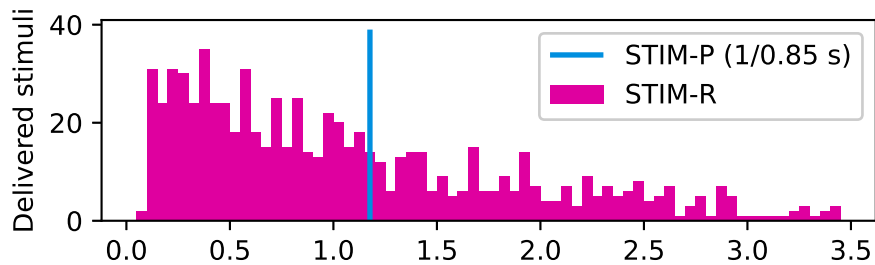**B**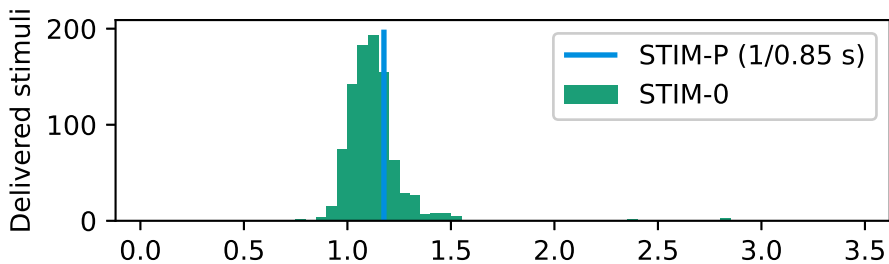**C**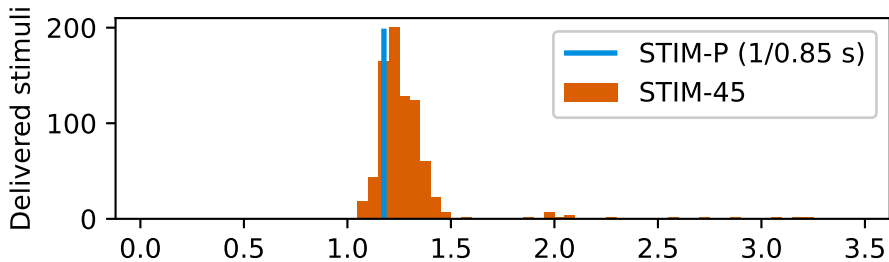**D**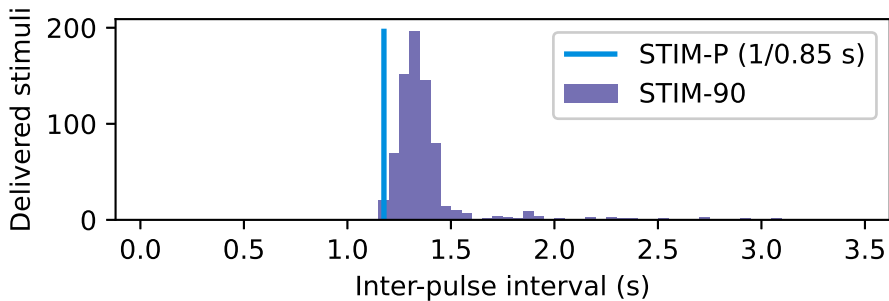

Supplement: S5 Fig — Histograms of time between delivered pulses in each condition: (A) STIM-R, (B) STIM-CL 0, (C) STIM-CL 45, and (D) STIM-CL 90. The bar of STIM-P is plotted as reference of the central frequency 0.85 Hz, the value of the probability distribution is higher than 5. The mode of the STIM-R interpulse is below 0.5 seconds. The statistical mode on the inter-stimuli increase as the target-phase of the closed loop increases. The statistical mode for STIM-CL 45 is the nearest to the rhythmic interval (STIM-P). (PDF) [file pcbi.1008758.s005.pdf]
