# Supplementary material for: Selection of stimulus parameters for enhancing slow wave sleep events with a neural-field theory thalamocortical model
Source: PLoS Comput Biol. 2021 Jul 30;17(7):e1008758. doi: 10.1371/journal.pcbi.1008758 (PMC8357165; doi:10.1371/journal.pcbi.1008758)
Supplement: S1 Table — The t-values and p-values for changes in power and occurrence of slow oscillation events by different stimulation case. The symbol ‘*’ indicates p < 0.01, and ‘w’ indicates that one stimulation case doesn’t accomplish the Shapiro test for normality. (PDF) [file pcbi.1008758.s006.pdf]

	$I^{(SO)}$		N_{SO}/min.	
	t-value	p-value	t-value	p-value
STIM-R SHAM	w0.0	6.2e-02	-9.1	*3.6e-5
STIM-P SHAM	69.4	*9.9e-08	-16.3	*3.5e-7
STIM-CL 0 SHAM	69.0	*2.1e-07	-10.3	*6.5e-5
STIM-CL 45 SHAM	69.4	*2.0e-07	-8.7	*2.2e-4
STIM-CL 90 SHAM	69.7	*1.1e-07	-12.7	*3.9e-6
STIM-P STIM-R	w0.0	6.2e-2	-5.4	*6.7e-4
STIM-CL 0 STIM-R	w0.0	6.2e-2	-3.9	1.7e-1
STIM-CL 45 STIM-R	w0.0	6.2e-2	-2.2	6.5e-2
STIM-CL 90 STIM-R	w0.0	6.2e-2	-3.1	1.5e-2
STIM-CL 0 STIM-P	-6.3	*8.8e-04	1.1	3.3e-01
STIM-CL 45 STIM-P	-4.5	*4.2e-03	1.7	1.5e-01
STIM-CL 90 STIM-P	0.7	5.0e-01	2.1	6.8e-02
STIM-CL 0 STIM-CL 90	-7.5	*3.1e-04	-0.6	5.7e-01
STIM-CL 45 STIM-CL 90	-5.6	*1.3e-03	0.1	9.2e-01
STIM-CL 0 STIM-CL 45	-2.8	2.5e-02	-0.6	5.8e-01
