# Supplementary material for: Selection of stimulus parameters for enhancing slow wave sleep events with a neural-field theory thalamocortical model
Source: PLoS Comput Biol. 2021 Jul 30;17(7):e1008758. doi: 10.1371/journal.pcbi.1008758 (PMC8357165; doi:10.1371/journal.pcbi.1008758)
Supplement: S2 Table — The t-values and p-values for changes in power and occurrence of sleep spindles events by different stimulation case. The symbol ‘*’ indicates p < 0.01, and ‘w’ indicates that one stimulation case doesn’t accomplish the Shapiro test for normality. (PDF) [file pcbi.1008758.s007.pdf]

	$I^{(SP)}$		N_{SP}/min.	
	t-value	p-value	t-value	p-value
STIM-R SHAM	-36.8	*4.9e-06	-8.8	*2.2e-05
STIM-P SHAM	-33.1	*3.2e-06	-8.5	*3.2e-05
STIM-CL 0 SHAM	-155.7	*1.8e-10	w0.0	4.3e-02
STIM-CL 45 SHAM	-147.8	*4.5e-11	-8.1	*4.1e-05
STIM-CL 90 SHAM	-128.0	*1.8e-13	-6.8	*1.5e-04
STIM-P STIM-R	-0.2	8.3e-01	-0.2	8.3e-01
STIM-CL 0 STIM-R	31.2	*6.3e-06	w6.0	6.8e-01
STIM-CL 45 STIM-R	31.3	*6.2e-06	1.1	3.1e-01
STIM-CL 90 STIM-R	31.4	*6.0e-06	1.6	1.6e-01
STIM-CL 0 STIM-P	w0.0	6.2e-02	w5.0	5.0e-01
STIM-CL 45 STIM-P	w3.0	3.1e-01	1.2	2.5e-01
STIM-CL 90 STIM-P	w0.0	6.2e-02	1.7	1.3e-01
STIM-CL 0 STIM-CL 90	-10.8	*2.6e-05	w0.0	4.3e-02
STIM-CL 0 STIM-CL 45	-7.5	*8.7e-05	w1.0	8.0e-02
STIM-CL 45 STIM-CL 90	-4.5	*2.6e-03	-0.6	5.7e-01
