# Supplementary material for: Selection of stimulus parameters for enhancing slow wave sleep events with a neural-field theory thalamocortical model
Source: PLoS Comput Biol. 2021 Jul 30;17(7):e1008758. doi: 10.1371/journal.pcbi.1008758 (PMC8357165; doi:10.1371/journal.pcbi.1008758)
Supplement: S3 Table — The t-values and p-values for changes in the probability of occurrence of slow oscillations and co-occurrences of events by different stimulation phase. Welch’s t-test applied after the confirmation of normality by Shapiro test. The ‘*’ indicates p-values <0.01. STIM-CL 0 number of spindles did not pass the normality test, and it neither did not pass the test with the SHAM condition, but is the highest value in the plot. (PDF) [file pcbi.1008758.s008.pdf]

	$P(SO)$		$P(C\|SP)$	
	t-value	p-value	t-value	p-value
STIM-R SHAM	122.9	5.5e-10	-8.8	*2.2e-05
STIM-P SHAM	w0.0	4.3e-02	-8.5	*3.2e-05
STIM-CL 0 SHAM	127.3	*1.2e-08	w0.0	4.3e-02
STIM-CL 45 SHAM	128.3	*8.0e-09	-8.1	*4.1e-05
STIM-CL 90 SHAM	127.7	*3.1e-09	-6.8	*1.5e-04
STIM-P STIM-R	w0.0	4.3e-02	-0.2	8.3e-01
STIM-CL 0 STIM-R	-5.6	*2.1e-03	w6.0	6.8e-01
STIM-CL 45 STIM-R	-1.1	3.2e-01	1.1	3.1e-01
STIM-CL 90 STIM-R	2.2	6.4e-02	1.6	1.6e-01
STIM-CL 0 STIM-P	w0.0	4.3e-02	w5.0	5.0e-01
STIM-CL 45 STIM-P	w3.0	3.2e-01	1.2	2.5e-01
STIM-CL 90 STIM-P	w0.0	4.3e-02	1.7	1.3e-01
STIM-CL 0 STIM-CL 90	-10.8	*2.6e-05	w0.0	4.3e-02
STIM-CL 0 STIM-CL 45	-7.5	*8.7e-05	w1.0	8.0e-02
STIM-CL 45 STIM-CL 90	-4.5	*2.6e-03	-0.6	5.7e-01
