# Supplementary material for: Selection of stimulus parameters for enhancing slow wave sleep events with a neural-field theory thalamocortical model
Source: PLoS Comput Biol. 2021 Jul 30;17(7):e1008758. doi: 10.1371/journal.pcbi.1008758 (PMC8357165; doi:10.1371/journal.pcbi.1008758)
Supplement: Attachment — Submitted filename: ResponseToReviewers.pdf [file pcbi.1008758.s010.pdf]

Reply to Reviewers: PCOMPBIOL-D-21-00190 - EMID:0457f61a9eba5baa *Selection of stimulus parameters for enhancing slow wave sleep events with a Neural-field theory thalamocortical computational model*

Felipe Torres, Patricio Orio, María José Escobar

March 22nd, 2021

We would like to thank the referees for their critical reading and pertinent concerns, as they have allowed us to improve our manuscript. We have prepared a revised version of our manuscript, which includes all the reviewers' observations. Bold fonts are used for the referees' comments and regular fonts for our reply. The text added or corrected is printed in blue in the new version of the manuscript.

The reference of the figures numeration is kept in the referees' comments. However, the figures order was changed. Fig 7 is now the Fig 1, and the other figures numeration were increased by one.

Reviewer #1

This paper uses an established corticothalamic neural field model to analyze the effects of various stimulation protocols on the occurrence of spindle (SP) and slow-oscillation (S) events, including the effects of stimulus timing relative to the SO. A key aim is to optimize stimulation to raise SO and SP power for potential applications to enhancement of memory consolidation. This work uncovers optimal regimes for stimulation and makes predictions that can be tested experimentally. It can thus be expected to contribute significantly to the advancement of the field of brain stimulation for memory enhancement. Overall, the paper is very strong and suitable for publication in PLoS-CB so long as the authors consider the points below.

We appreciate the reviewer for these comments about our contribution. In the following, we will address the comments one by one.

My comments are: 1) The authors should clarify a little more explicitly in the Introduction that their working hypothesis is that SOs and SPs enhance memory, rather than simply having a common cause with memory enhancement.

A brief clarification is now in line 84 relating the enhancement of memory in further experimental works. Our simulation results only concern the experimental protocol enhancing SO and SP events.

2) line 115: what are the units of phi_nˆ(0)?

We apologize for the omission. The units of $\phi_n^{(0)}$ are s^{-1}. This is now indicated in the methods and added to line 141.

3) table 1 and throughout the MS: physical units should be written in roman typeface, not italic. What is "std"?

The physical units are now in roman font. The abbreviation "std" refers to standard deviation, but to avoid

confusion, we have changed it to "SD," and it is defined in the caption of Table 1.

4) p6: It might be worth mentioning that the SO and SP events are "evoked responses" - i.e., use this terminology because this is a large field and may attract interest from that community. This might be good to have as a keyword.

We appreciate the suggestion, but we consider it not fully adequate to associate SO and SP events as evoked responses. In the first place, these events occur even without stimulation. The first part of the results searches for the model parameters generating spontaneous SO and SP events. Secondly, not every applied stimulus results in an 'evoked' event, but rather the increase in the events probability results from the interaction of the evoked responses and the ongoing activity.

5) Table 2 needs a caption.

The table now contains a caption describing the contents of each column.

6) Fig 3 caption - "trapeze" should be "trapezium"

Thank you for noting our mistake. We have noted that in American English (which is the style we use throughout the paper), the word should be "trapezoid," and for the "half trapezium," the term should be "rectangular trapezoid." Both the caption in Fig 3 (now Fig 4) and Table 2 have been corrected.

7) line 221: I'm not sure what was intended by the word "overpasses". Should it be "outperforms" or something similar? This word appears later in the MS too - e.g., line 558 where it perhaps should read "The trigger waits for the phase 1.9 or 342 degrees to be reached before ..."

Indeed, the word "outperforms" fits better with our intended message on line 256. The replacement of "overpasses" with "outperforms" was also made in lines 261 and 263. In the line 471, "overpasses" was replaced by "surpasses." Moreover, in the line 600, the replacement was with the word "exceeds." Finally, the recommended phrase was replaced in line 640.

8) line 294: should "notorious" be something like "notable". I'm sure that "notorious" is not what the authors intended.

The line 364 now reads 'notable'.

9) line 415. The notation with b epsilon b will probably be a bit confusing to some neuroscientists. Perhaps just avoid it with a slight rewriting. Maybe say b epsilon p and mention the meaning of the epsilon symbol and state what elements are in the set p.

Line 490 now contains "with a, b being elements of the set of neural populations $\mathbf{p} = e, i, r, s$". This explanation, that was previously indicated in line 495, has been removed. Also, the notation used for the set of populations in Eq 1 changed from **b** to **p**.

10) Eq 1 - first term on left has two sets of arguments (r,t).

The additional set of arguments was removed from the first term on the left of Eq 1.

11) the subscript max in Eq 2 and elsewhere should be in roman font - it's a mathematical function. Likewise atan on line 563

The subscript "max" in Eq. 2 and elsewhere is now in roman font, including the missing subscript in Table 1. Besides, the mathematical function 'atan' is also now in roman font in line 636.

12) line 435 - "sub-indexes" should perhaps be subscripts

Line 510 now says "subscripts".

13) line 476 - write as 10ˆ-4 (i.e., with a superscript) rather than in computer notation 1e-4; likewise on line 506 and elsewhere

The notation in lines 554, 584, 587, 596 and 625 was changed to the format with a superscript.

14) 488 - first argument of phi_e should be r

The first argument of ϕ_e now is $\mathbf{r}$ in line 569.

15) line 534 - please add a few words of description of the Morlet wavelet.

The Morlet wavelet has now a brief description in line 615.

16) I suggest replacing "aleatory" by "random" throughout the paper - the former word does exist in English, but is exceedingly rare, despite its similarity to the Spanish word for "random" (I see that the authors are from a Spanish-speaking country).

The word 'aleatory' is now replaced by random throughout the paper.

17) In some places the paper could have been made clearer by using the present tense for the present work and past tense for past work - the authors use the reverse convention for some reason.

Thanks for the observation, we reviewed the article to make it clearer with the use of the suggested verbal tense.

The used convention along the paper when writing about the present work is: Results section uses the present tense, but we used the past tense when we are indicating methodological steps or procedures (i.e. "things that were done"). Discussion section uses the present tense. Finally, Methods section is written in past tense.

18) Title: The model is not a "computational" model. Computational methods are used to analyze it, but it is not formulated in terms of brain computations.

The word 'computational' is now removed from the title. As neural field theory is related to brain computations, 'computational' is redundant in the title. The term 'thalamocortical model' is enough to describe the contribution of this article.

19) Fig 4D. Are we seeing a leveling off here because it is impossible to squeeze in more than about 20 SOs/min without them overlapping.

Indeed, thanks for the observation. A commentary about this leveling off is now in line 225.

20) The caption of Fig 6 needs to refer explicitly to the curves at top.

The caption of Fig 6 (now Fig 7) is now modified with "averaged time series at the top".

21) Supporting Information needs at least a brief preamble to explain what materials are included.

A brief preamble is now included in the Supporting Information section.

22) It is much easier for the referees if the figures are placed where they are used in the MS, rather than at the end. If the journal requires them to be at the end they can be put there too or moved there upon acceptance.

We understand the frustration of the reviewer and the difficulty of the revision process. However, we are just following the journal's guidelines. For this second submission, the tracked-changes version of the manuscript now contains the figures in-line. In contrast, the clean version follows the guidelines.

Reviewer #2

This paper gives a comprehensive description of the authors' implementation, parameter selection, numerical simulation and data analysis of their computational model, namely eirs-NFT model, based on the Neural Field Theory (NFT). The paper suggests that a decreasing ramp of 50 ms duration is the most effective amongst six stimulus shapes, and that the best effectiveness is achieved when the stimulus impulse is delivered in a closed-loop configuration targeting at the zero phase of the ongoing slow oscillation event.

Although the main contribution of the paper is its suggestion on the design of stimulus in real-

life sleep experiments, the event detection methods are non-canonical from an experimentalist's perspective. In "Event detection in the time domain", the authors acknowledged this limitation, and showed their endeavour in reducing any chance of false detection for frequency drifts or amplitude variability. However, it would still be interesting to know what is the interval of amplitudes (even with arbitrary units) detected for slow-oscillation detections. For spindles their detection is more straightforward. A graph summarising the events detected would be ideal. Even though it may not be accurately comparable to real-life results, it could offer more convincing evidence that their non-canonical method managed to detect events as expected.

The interval of amplitudes of detected slow-oscillations is -0.025 - 0.025 s^{-1} around the mean propagation field. Following the reviewer's suggestion, the new panels of Fig 3 (E and F) show some details about the detected events. Specifically, histograms of the time duration and the peak-to-peak amplitude for SP and SO events.

The paper's second major contribution is its deployment of the eirs-NFT model in the particular sleep stage. However, despite a brief mention in "Introduction" and a detailed recipe in "Materials and methods/Large-scale brain model: Neural Field Theory", a description of the model is missing from "Results/Selection of model parameters for SWS stage", making the XYZ space on page 4 and in Fig 1A and the chosen values of model parameters in Table 1 decontextualised and difficult to understand unless a reader is very familiar with previous works [24, 25] by other authors. The novelty of this work could thus be undervalued due to a lack of appreciation of the model and its parameters. At least the authors could give some verbal description in the text or in Table 1 to give readers (those unfamiliar with the model, or even the theory) some intuition why these parameters and the axes XYZ are important in the computational model, and how they are related to real-life experiments.

In this revised version, the results section now includes a brief model description, together with Fig 1 showing the model architecture and the principal modules. Besides, we enlarged the caption of Table 1, including a description of the model parameters.

Miscellaneous on formatting: Last line in Fig 1 caption on page 6: the subscriptions of G_ee and G_ei should be italic

The indicated subscriptions in the last line of the new Fig 2 (previous Fig 1) caption are now in italics. We also checked the entire document to homogenize the notation.

Fig 4: One can probably guess the horizontal axes of A, B, C share the same labels as D, E, F, but it was not an easy guess for me because the scales are different. Or I guessed wrongly.

Indeed, the horizontal axes label $I^{(SO)}$ is the same for A and D, $I^{(SP)}$ for plots B and E, and $P(SO)$ for plots C and F. This indication is now added in the caption of Fig 4 (now Fig 5).

Line 273 on page 10: 'Tab 3' is inconsistent with previous notations.

Following the notation in the paper, 'Tab 3' is now replaced with 'Table 3' (now Table 2) in line 329.

Line 315 on page 11: missing space before 'In our simulations, ...'

We included a space before this sentence.

Line 415 & 435 on page 13 & 14: missing space before '7B'

A space is now added before '7B' (now '1B') in the indicated positions.

Line 571 on page 18: obvious

The redundant phrase is now deleted.

Reviewer #3

Major issues:

Although the authors have addressed a crucial issue in this field, the neurobiological significance of this study is not clear without supportive evidence to confirm the authors' predictions.

We thank the reviewer for raising this concern. Nevertheless, to confirm the prediction indicated in this contribution falls out of this article's scope. We decided to focus on the simulation to provide a computational framework to better design real-life sleep experiments.

Minor issues: Introduction The citations do not reflect this field well. I would suggest to cite papers/reviews written by Steriade, Buzsaki, and other pioneers in order to provide an appropriate context to readers.

An additional paragraph is now in the introduction about the sleep EEG rhythms origins on the thalamocortical system.

Results Because the Methods section comes later, provide the technical definition of SOs and SPs briefly.

Following the reviewer's recommendation, we now provide a technical definition of SO and SP in the results section *Enhancement of SWS events on the eirs-NFT model.*

Figure 1A requires more explanation. For example, scales are missing for the example EEG traces. It is not clear when SPs appear.

The caption of Fig 1A (now Fig 2A) now contains the missing scales, and the y-axis ticks are more notorious. Besides, the filtered signal root-mean-square value at the bands of interest of the events is now above the time-series excerpts of the same figure, indicating the presence of activity at those frequency bands. Even if the root-mean-square values do not indicate individual SPs' appearance, they indicate the temporal rising and decaying of activity at both bands, emphasizing the differences between the presented excerpts rather than the temporal marks of detected spindles.

In Fig. 3, what are the biological justification of all these pulses? Note that if this mean-field model assumes the auditory pathway, there are multiple auditory nuclei before signals reach the medial geniculate body. Thus, it is hard to believe that any sensory (acoustic) stimuli will appear as any of these. I would suggest that the authors should address how noises affect all measures at least.

In this article, we tested different input stimuli varying the shape, amplitude, and time width of the input pulses on the thalamic relay nuclei. According to Fig. 1C, the input pulse is combined with noise and then convolved with a Difference-of-Gaussian filter to feed the thalamocortical neural field. One of the advantages of the eirs-NFT model is its capability to simulate evoked response potentials (ERPs) from the input pulses (Zobaer et al. 2018), representing the multiple auditory nuclei processing the input signal. This is regardless of the absence of the sensory pathway from the cochlea to the thalamus.

In the model, the inputs are represented by firing responses correlated in time with the sensory pulse and with a firing rate proportional to the stimuli magnitude. The shape of the stimulus represents different neural population responses, and that could be differentiated by their selectivity of stimulus parameters, as the carrier frequency. A rectangular shape assumes a population with a constant firing rate. On the other hand, the decreasing ramp and the rectangular trapezoid indicate a short-term adaptation to the applied stimuli (Smith and Zwislocki 1974). The other shapes are less physiologically related to possible mean firing responses for a sensory stimulus, but we tested them to test differences in the shapes. We also assumed that the receptive field, represented by a Difference-of-Gaussians, is a good enough spatial representation of the population activity for their preferred stimuli (Krishna 2000, Wang 2018).

Following the reviewer's comment, we now include a supplementary figure showing how the different pulse shapes generate different ERPs at the cortex (new Fig S1). Moreover, we also added a new paragraph summarizing this answer in the line 337.

Krishna, B. Suresh & Semple, Malcolm N. Auditory Temporal Processing: Responses to Sinusoidally Amplitude-Modulated Tones in the Inferior Colliculus. Journal of Neurophysiology. 2000; 84:1, 255-273

Smith R. L., & Zwislocki J. J. Short-Term Adaptation and Incremental Responses of single Auditory-Nerve Fibers. Biol Cybern. 1975; 17, 169-182

Wang Xiaoqin. Cortical Coding of Auditory Features. Annual Review of Neuroscience. 2018; 41:1, 527-552 https://doi.org/10.1146/annurev-neuro-072116-031302

Zobaer, M.S., Robinson, P.A. & Kerr, C.C. Physiology-based ERPs in normal and abnormal states. Biol Cybern. 2008; 112, 465–482. https://doi.org/10.1007/s00422-018-0766-x

In Fig. 4, only rectangular pulses were applied. How about other pulses?

The results of Fig 4A-C (now Fig 5A-C) have the intent of testing the pulse duration as a dependent variable. The advantage of using the rectangular pulse, instead of any other pulse shape, is that it keeps the amplitude constant along the pulse duration. A brief comment about this is now added in the line 211.

In Table 3, the bottom left (or top right) half is redundant.

Indeed, as the reviewer points out, the matrix showing the statistical difference comparing two stimulation conditions is symmetric. Following the reviewer's recommendation, the p-values are only present in half of the table.

In Figure 6 and Table 3, although the outcomes of the close-loop stimulation are intriguing, the authors did not provide any explanations why these are the case. Further analysis will help to better understand (1) why the 0 deg stimulation induced more SO/SP couplings and (2) why 45/90 deg stimulation changed the SP distribution. In addition, what are impacts of this shift on the thalamocortical circuit dynamics?

The outcomes of Figure 5 and Figure 6 (now renamed as Figure 6 and Figure 7) can arise from the different inter-stimuli interval distributions produced with the stimulation at different target phases. The $0°$ stimulation delivers pulses at a smaller inter-pulse than the $45°$ and $90°$ stimulations. More time is required to find again the target-phase if the stimulus is applied farther from the rising zero-cross point. Then, in the same stimulation time window, more pulses in $0°$ condition increase the number of events and provide more opportunity to SO/SP couplings. The inter-stimuli intervals distributions are now in the supplementary S8 Figure and the number of delivered pulses in the caption of Figure 7.

Input stimulus modifies cortical and thalamus activity. If the stimulus is given when the basal activity is close to reaching a maximum (phase $90°$), the evoked response will shift the timing of the peak response because the activity - which was about to reach zero slope - increases again. Then, the following maximum is delayed. On the other hand, the stimulus generates a burst activity at the thalamus that could last enough to cause cortical spindles. Both stimulus effects could justify the difference of SPs timing distribution in relation with the SOs.

This analysis is now part of the results in line 288, and of the discussion in line 322.

Discussion Before discussing technical issues, it would be better to summarize key observations first.

Following the reviewer's suggestion, the discussion now starts by summarizing the research findings.

Neurobiological significant and limitations of this study must be discussed if any.

One of the model limitations is the assumption of sleep steady stage along all the 910 seconds of each simulation. Also, the pulses firing rate amplitude was chosen to surpass the noise variation. Still, there is no declared neurobiological relation with the firing rates produced by certain sound pressure levels of the sensory pulse. These limitations are now highlighted in lines 439 and 456.

Reviewer #4

SUMMARY

The article by Torres et al is a comprehensive investigation into the promotion of physiological sleep signatures using rhythmic sensory stimulation, within the framework of the corticothalamic neural field model of Robinson et al. The authors began with the reduced three-dimensional 'XYZ' parameter space of the linearized, spatially uniform steady state version of the model.

Linear interpolating between previously identified N2 and N3 points in the XYZ space identified a point in parameter space showing both slow oscillation (SO) and sleep spindle (SP) events (peaks in the linear power spectrum). The authors then moved to the full nonlinear model, consisting of 'alpha kernel' synaptic response functions, sigmoidal wave-to-pulse transfer functions, a variety of input waveforms entering the thalamic relay population, and spatiotemporal wave propagation across the cortical sheet (here simply a 2D grid) following a damped wave equation. The spatial dimension is then collapsed down by averaging over space, yielding a scalar time series that is the principal object of study for the rest of the paper. The key result is that co-occurrence of SO and SP events was maximized by 50ms decreasing ramp pulses, delivered at the zero phase of filtered ongoing activity.

RECOMMENDATION

Rhythmic sensory and electromagnetic stimulation for promoting sleep is an emerging area of study that has received considerable attention in recent years, both from academic science and commercial biotech. Work in this area has however been almost entirely atheoretical, outside of generic signal processing. As such, the in-depth physiologically-based investigation presented in this submission is a valuable, and fairly original, contribution to both the computational neuroscience and sleep physiology literature.

I am therefore happy to recommend the paper for publication in PLoS Computational Biology, provided the authors can address the following issues:

We appreciate the reviewer's comments. The issues raised by the reviewer are here addressed in detail.

REVISIONS

Major points

Analytic vs. numerical power spectrum:

A central tenet of the study methodology is the selection of model parameters to study using the linear algebraic power spectrum model, and the further investigation of that point in parameter space with numerical simulations in the full PDE model. This is a very powerful general approach. However I think it is important to demonstrate correspondence between the analytic and numerical power spectra, for the selected parameters, by producing spectra with each method, and overlaying on the same graph.

Relatedly: the power spectrum in Figure 2D seems very noisy. Indeed this is unsurprising, as the legend description indicates this is computed from only 15 seconds of data, out of the 910 seconds of simulation. Presumably, the effect of interest - enhancement at SO and SP frequency bands - would also appear, more strongly, in a power spectrum computed over several minutes, rather than 15 seconds.

I would suggest to put both of these (analytic vs. empirical spectra, and a longer-run simulation) in a new Figure 2, and increment the figure numbers for Figure 2 onwards in the current text. But I leave it to the authors to decide how best to address this issue.

Figure 2D (now Fig 3D) now contains the analytical power spectrum. The spectrum is re-normalized by the max value of the numerical power spectrum. Also, the numerical power spectrums are now calculated with the 910 seconds of simulation.

Minor points

Implementation details:

Apart from the usage of Matlab fsolve for solution of Eqn. 4, there is not, so far as I have seen, a description of the model implementation in the methods. What programming language? Operating system?

The model was implemented on Matlab, and it is available on the repository https://github.com/mjescobar/SWSNeuralField. The simulations were performed on a computer running the operating system Ubuntu Server 18.04 with a double Xeon E5-2630 v4 processor. This information is now given in line 556.

Reproducible code:

The questions in the opening comment boxes indicate that 'all data will be made available'. But there are no further details on this. Presumably this is actually referring to code, which is the main outcome of this purely numerical simulation study. Please clarify what will be placed in the public domain, and where (github repository? Figshare? Supplementary file?)

The model implementation code, the scripts for the simulations, and the paper figures' scripts are now in a Github repository, which is now also indicated in the manuscript.

The Github repository is: https://github.com/mjescobar/SWSNeuralField.

Boundary conditions:

Please give some detail on the boundary conditions used for the numerical simulations. The Robinson model has been studied on planar, toroidal, and spherical domains. The relationship between the spatial damping rate and boundary conditions is a major emphasis in this work (e.g. Robinson et al. 1997), albeit with the general conclusion that it is not hugely important.

The used boundary conditions are now expressed in the line 555.

Movie:

Given that activity is simulated across the spatial patch, it would be a nice touch to add a movie to the supplementary information, showing activity in the patch.

The patch activity movie is now presented in the supporting information and stored in the code repository at https://github.com/mjescobar/SWSNeuralField/blob/main/analyze/plotting/SWSVideo.mp4.

Figure 2B:

Please reverse the y axis ordering for figure 2B. It is confusing having frequencies descending with increasing Y in a time-frequency plot.

Thanks for the suggestion. We have modified the Figure 2B (now Figure 3B) so that the frequencies now go up now in the y axis.

Figure text locations:

To whomever it concerns: it is very frustrating to have figures at the end of a text, with legends in the main text. Strongly recommend just put the figures in-line in the future in for-review manuscripts.

We share the frustration of the reviewer and the difficulty of the revision process. However, we are just following the journal's guidelines. For this second submission, the tracked-changes version of the manuscript now contains the figures in-line. In contrast, the clean version follows the guidelines.
