# Supplementary material for: Selection of stimulus parameters for enhancing slow wave sleep events with a neural-field theory thalamocortical model
Source: PLoS Comput Biol. 2021 Jul 30;17(7):e1008758. doi: 10.1371/journal.pcbi.1008758 (PMC8357165; doi:10.1371/journal.pcbi.1008758)
Supplement: Attachment — Submitted filename: ResponseToReviewers.pdf [file pcbi.1008758.s011.pdf]

Reply to Reviewers: PCOMPBIOL-D-21-00190 - EMID:0457f61a9eba5baa
Selection of stimulus parameters for enhancing slow wave sleep events with a Neural-field theory thalamocortical computational model

Felipe Torres, Patricio Orio, María José Escobar

May 17th, 2021

We would like to thank the referees for their critical reading and pertinent concerns, as they have allowed us to improve our manuscript. We have prepared a revised version of our manuscript, which includes the last reviewers' observations. Bold fonts are used for the referees' comments and regular fonts for our reply. The text added or corrected is printed in blue in the new version of the manuscript.

Reviewer #1

The authors have dealt thoroughly with my previous comments and the paper is now suitable for publication.

I do want to stress that realistic predictions such as these are highly biologically relevant - they will provoke quantitative tests by experimentalists. It is certainly not necessary for the authors to do these experiments themselves - which would be a very unusual situation in more mature branches of science and engineering, where the need for specialization of theorists and experimentalists has been recognized for centuries.

Thanks for your time in the revision of our work. We appreciate your support for theoretical work in biology.

Reviewer #2

We appreciate the authors' responses and changes. Two follow-up minor issues:

1. Why the unit of the amplitude in the new panel 3F is s^{-1}? Apparently this is due to the choice of arbitrary units, but this might be misleading to many readers.

Thanks for your time in the revision of our work.

The events are detected in the model's outcome, $x(t)$, obtained from the spatial mean of ϕ_e, subtracting its spatiotemporal average (See Fig. 1C). Although $x(t)$ has similar dynamics to EEG recordings, it actually represents the mean firing rate with units s^{-1}. We made an additional annotation in the caption of Fig. 3, explicitly indicating $x(t)$ as a firing rate variable.

2. This recent experimental paper (Navarrete et al 2020)[https://doi.org/10.1093/sleep/zsz315] might be interesting to the authors, as they were seemingly suggested the same timing for delivering stimulus during sleep.

We thank the reviewer for the literature reference. We have now included Navarrete et al., 2020 in the discussion

(line 451), linking their result with our theoretical approach.

Reviewer #3

The authors have addressed my initial concerns thoroughly. As a result, the revised manuscript has been improved significantly. Although the neurobiological significance of this elegant study is still an open question, I strongly believe that the current version is suitable for a publication.

We thank the reviewer for your time in the revision of our work. We agree in that further experimental works will provide more significant connections with the simulated neurobiological phenomena.

Reviewer #4

The authors have addressed all my concerns and recommendations comprehensively. No further comments. Great paper, looking forward to seeing it published.

Thanks for your time in the revision of our work. We appreciate your supporting commentary.
